# Biaxial Non-Resonant Electromagnetically Driven Scanning Micromirror with Large Aperture

**DOI:** 10.3390/mi16060610

**Published:** 2025-05-23

**Authors:** Tong Wang, Yu Jian, Chen Liu, Manpeng Chang, Xin Wang, Weimin Wang

**Affiliations:** 1 Key Laboratory of Optoelectronic Technology and Systems, Ministry of Education, Chongqing University, Chongqing 400044, China; tong.w@stu.cqu.edu.cn (T.W.); jianyu@stu.cqu.edu.cn (Y.J.); chenliu@stu.cqu.edu.cn (C.L.); changmanpeng@stu.cqu.edu.cn (M.C.); wangxin716@stu.cqu.edu.cn (X.W.); 2 Defense Key Disciplines Laboratory of Novel Micro-Nano Devices and System Technology, Chongqing University, Chongqing 400044, China; 3 College of Optoelectronic Engineering, Chongqing University, Chongqing 400044, China

**Keywords:** electromagnetically driven scanning micromirror, biaxial non-resonant scanning mode, large aperture

## Abstract

To address the challenges of small aperture size, limited scanning angles, and high fabrication costs in existing scanning micromirrors, this paper proposes a large-aperture biaxial electromagnetically driven scanning micromirror. The scanning micromirror utilizes a stainless-steel mirror structure and an actuation structure composed of arc-shaped permanent magnets (NdFeB 52), iron cores, and copper coils. By optimizing the magnet layout and coil design, it achieves large optical scanning angles in biaxial non-resonant scanning mode. Experimental results demonstrate that the optical scanning angles reach 61.4° (x-axis) under a DC driving current of ±18.1 mA and 61.1° (y-axis) under a DC driving current of ±25.2 mA with an effective mirror aperture of 9.54 mm × 10 mm. The resonant frequencies are 89 Hz (x-axis) and 63 Hz (y-axis). Experimental results verify the feasibility of biaxial independent control in non-resonant scanning mode. The design is fabricated using a low-cost computer numerical control (CNC) milling process and exhibits application potential in fields such as LiDAR, projection display, and optical communication, providing a novel approach for performance optimization of large-aperture scanning micromirrors.

## 1. Introduction

Scanning micromirrors are miniature precision optical devices capable of controlling the position and direction of light beams with micrometer-level precision. Currently, scanning micromirrors are primarily applied in fields including projection displays [1,2,3], optical coherence tomography (OCT) [4,5,6], two-photon microscopy [7], 3D optical tracking [8], light detection and ranging (LiDAR) [9,10,11,12,13,14], and free-space optical communication [15,16].

Compared to conventional galvanometers, scanning micromirrors offer advantages such as compact size, high speed, and high reliability [17,18]. The relatively low fabrication cost of scanning micromirrors enables mass production, making them competitive in commercial applications [19]. The primary actuation methods for scanning micromirrors include electrostatic actuation, piezoelectric actuation, electrothermal actuation, and electromagnetic actuation. The fabrication processes of electrostatically actuated scanning micromirrors are compatible with existing technologies. However, the complex circuit design and high actuation voltage limit the miniaturization of scanning micromirrors [20,21]. Electrothermally actuated scanning micromirrors offer large deflections at relatively low actuation voltages. However, the response speed is limited by thermal expansion, which affects the dynamic performance of the micromirrors. Piezoelectrically actuated scanning micromirrors are restricted by their material properties, and the manufacturing process is relatively complex [22]. Compared to other actuation methods, electromagnetic actuation can offer a large driving force under the interaction between permanent magnets and coils to achieve a large deflection and high-speed scanning. Also, electromagnetic actuation has been widely adopted in scanning micromirror driving systems [23,24,25]. Thus, this study used the advantages of electromagnetic actuation’s driving force to design and implement scanning micromirrors.

Conventional MEMS scanning mirrors are limited by silicon-based fabrication processes, typically with aperture sizes below 10 mm. The small aperture size of scanning micromirrors can limit their ability to collect sufficient scattered light in LiDAR, projection display, and OCT. For large-aperture MEMS scanning micromirrors, increasing aperture sizes result in significantly diminished application advantages due to prohibitive fabrication costs and process constraints. However, large-aperture scanning micromirrors not only tolerate high optical power but also maximize the utilization of light energy [26]. Also, some applications need large-aperture scanning micromirrors, such as automated guided vehicle (AGV) and robot navigation and obstacle avoidance [9]. Considering their application requirements and cost-effectiveness, developing low-cost large-aperture scanning micromirrors is necessary. Currently, researchers have recognized the broad application prospects of large-aperture scanning micromirrors. For instance, Songtao Liu et al. developed a novel permanent magnet structure (PMS) to address torque insufficiency in electromagnetic actuation through a novel permanent magnet-driven configuration. The optimized PMS enables an MEMS scanning micromirror with an 8 mm diameter to achieve an optical scanning angle of 30° × 25° under resonant scanning mode [27]. Siyuan He et al. developed a uniaxial scanning system based on a flexible printed circuit board (FPCB) micromirror. With a mirror size of 25 mm × 50 mm, this electromagnetically driven scanning micromirror achieves an optical scanning angle of 60° under non-resonant scanning mode [9]. Although large-aperture scanning micromirrors have attracted more research attention, it is hard to balance scanning frequency, mirror size, and optical scanning angles. Thus, developing a large-aperture scanning micromirror with wide optical scanning angles could provide novel strategies for advancing research.

Currently, MEMS processes are mainly used to fabricate micromirrors, offering advantages such as a high scanning frequency, light weight, and low power consumption [17,18]. However, the silicon-based micromirror manufacturing process requires precision microfabrication equipment and complex process steps. While single-crystal silicon is a high-strength material [28,29], its fatigue strength decreases as the structural size increases [30]. Due to the inherent fragility of single-crystal silicon, fabricating large-aperture micromirrors remains challenging. With advancing research in scanning micromirrors, researchers are not limited to silicon-based materials and have explored alternative materials, including metals [31,32,33,34] and polymers [14,24]. Corresponding fabrication processes have also evolved, such as 3D printing [14,25], electrical discharge machining (EDM) [35], and CNC milling [34].

For instance, Ching-Kai Shen et al. developed a 3D-printed scanning micromirror with a mirror size of 20 mm × 20 mm featuring a short fabrication cycle and low production cost. However, this design is limited to uniaxial scanning, achieving a full optical scanning angle of 29.6° under a 12 V DC driving voltage [25]. Liangchen Ye et al. fabricated a Ti-alloy based uniaxial electromagnetically driven scanning micromirror via EDM, with a mirror diameter of 12 mm and an optical scanning angle of 26° at a resonant frequency of 1.4 kHz [35]. Hyun Yoon et al. presented a piezoelectric metal scanning micromirror using CNC machining to construct a stainless-steel structural layer, with a 5 mm diameter gold-coated silicon mirror plate as the reflective surface. The experimental results reveal that this micromirror achieves a uniaxial optical scanning angle of 44° with a sinusoidal input voltage of 200 Vpp [34]. These novel fabrication methods have diversified the development of scanning micromirrors. In this work, we propose a novel driving structure for scanning micromirrors. Using stainless steel as the mirror plate material simplifies fabrication steps, thus improving production efficiency and profit potential.

In the field of biaxial scanning micromirrors, there are three common scanning modes: biaxial resonant, uniaxial resonant and non-resonant, and biaxial non-resonant modes. Most micromirrors operate in biaxial resonant scanning mode, while some operate in uniaxial resonant and non-resonant scanning mode. However, few works have been reported on the biaxial non-resonant scanning mode, and existing research in this area is limited by aperture size. For example, Bu Hyun Shin et al. developed a biaxial electromagnetically driven scanning micromirror with a size of 8 mm × 8 mm, achieving optical scanning angles of 90° and 50° under biaxial resonant frequencies of 60 Hz × 60 Hz. In biaxial non-resonant scanning mode, this micromirror achieves optical scanning angles of 15.7° × 16.2° [36]. Similarly, Caglar Ataman et al. fabricated a 4 mm × 4 mm electromagnetically driven scanning micromirror, which achieves optical scanning angles of 32° × 32° under biaxial non-resonant scanning mode [37]. It is more difficult to achieve larger scanning angles in biaxial non-resonant scanning mode than in biaxial resonant scanning mode, mainly because of torque limitations. In contrast to the biaxial resonant scanning mode, the biaxial non-resonant scanning mode has no inherent frequency constraints. This allows for precise control of deflection angles and motion trajectories through external driving signals, enabling higher-precision positioning and scanning. In biaxial non-resonant scanning mode, the motion frequency and amplitude of scanning micromirrors can be adjusted to adapt to diverse application scenarios. Because of the advantages of operational modes and control precision in biaxial non-resonant scanning mode, developing a biaxial electromagnetically driven non-resonant scanning micromirror is imperative.

In this paper, we propose a large-aperture biaxial electromagnetically driven scanning micromirror and present its design and experimental validation. The remaining sections are organized as follows: Section 2 details the structural design. Section 3 introduces the modeling and simulation framework. Section 4 elaborates on the experimental results. The discussion and conclusions are presented in Section 5 and Section 6.

## 2. Design

**Figure 1:**
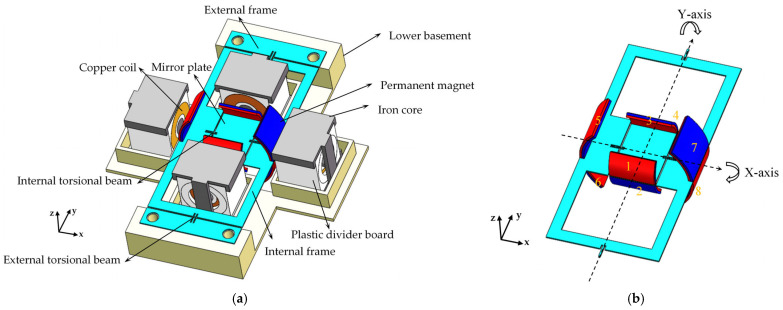
(**a**) The schematic structure of the proposed biaxial electromagnetically driven scanning micromirror. (**b**) The mirror structure of the scanning micromirror.

Figure 1a illustrates the schematic structure of the scanning micromirror, demonstrating the design concept of this work. The mirror structure is fabricated from stainless-steel (UNS S30400) and mounted on the lower basement, as shown in Figure 1b. Arc-shaped permanent magnets (NdFeB 52) are attached to the top and bottom surfaces of the mirror plate and internal frame, and copper coils and iron cores are distributed around the permanent magnets. The internal torsion beams enable the mirror plate to move around the x-axis, while the external torsion beams allow the mirror plate and the internal frame to move around the y-axis.

Without permanent magnets, the mirror plate has dimensions of 15 mm × 10 mm. Permanent magnets with opposing polarities are attached to the top and bottom surfaces of the mirror plate, labeled as magnet pairs 1–2 and 3–4 in Figure 1b. Similarly, permanent magnets with opposing polarities are attached to the top and bottom surfaces of the internal frame, labeled as magnet pairs 5–6 and 7–8 in Figure 1b. For clarity, the following analysis focuses on the interaction between permanent magnets on the mirror plate and copper coils, while the interaction between permanent magnets on the internal frame and copper coils operates on the same principle and thus is not discussed here. The arc-shaped design of permanent magnets on the mirror plate provides sufficient space for their movement. To achieve this, the external diameter of permanent magnets on the mirror plate ($D_{\mathrm{eim}}$) should match the length of the mirror plate ($L_{m}$).

In the actuation structure design, arc-shaped permanent magnets (NdFeB 52) 1–4 are attached on the mirror plate, while the coils are arranged on both sides of the mirror plate. When the driving current is applied to the copper coil, a vertical magnetic field is generated according to Ampere’s law, which applies an attractive or repulsive force to the magnet depending on the current direction. When a forward current is applied to the left coil (Co_A_), permanent magnet 1 experiences an attractive force while permanent magnet 2 experiences a repulsive force, causing the left mirror plate to move downward. When a reverse current is applied to the right coil (Co_B_), permanent magnet 3 experiences a repulsive force while permanent magnet 4 experiences an attractive force, driving the right mirror plate to move upward. Similarly, when a reverse current is applied to the left coil (Co_A_) and a forward current is applied to the right coil (Co_B_), the left mirror plate moves downward and the right mirror plate moves upward, causing the mirror plates to deflect in opposite directions.

To enhance the electromagnetic torque, the iron cores are incorporated into the copper coils. Since the iron cores inherently generate magnetic attractive force with the permanent magnets, their installation requires precise alignment: the center of the iron core thickness should coincide with the center of the mirror plate thickness. This ensures that the top and bottom permanent magnets experience balanced magnetic attractive forces, keeping the mirror plate horizontal when no current is applied.

**Figure 2:**
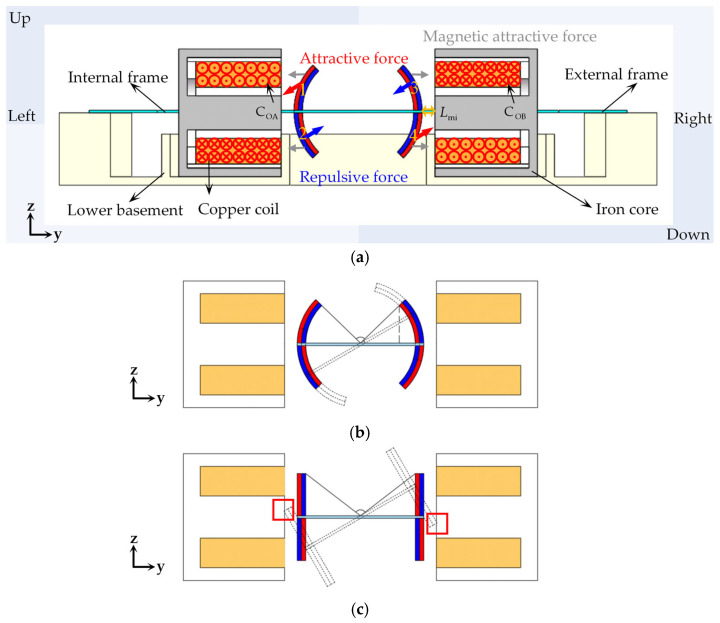
(**a**) The operation principle of the designed micromirror. (**b**) The deflection process of rectangular permanent magnets. (**c**) The deflection process of arc-shaped permanent magnets.

As shown in the red box in Figure 2b, when the rectangular and arc-shaped permanent magnets in Figure 2b,c rotate through the same angle, the rectangular ones will contact the copper coils. Therefore, during the design phase, the distance between the permanent magnets and the copper coils ($L_{\mathrm{mi}}$) should be taken into account, and sufficient space needs to be reserved for the movement of the permanent magnets. In contrast, the arc-shaped permanent magnet design eliminates this concern. The arc-shaped permanent magnets offer the advantage of minimizing the distance between the permanent magnets and the copper coils. However, the drawback is that the design of arc-shaped permanent magnets obstructs part of the light propagation. The maximum light propagation angle depends on the curvature of the permanent magnets. In this work, each arc-shaped permanent magnet is 45°, resulting in a maximum light propagation angle of 90°. With the arc-shaped permanent magnets installed, the effective aperture of the mirror plate can reach 9.54 × 10 mm. Detailed dimensions of the mirror structure and actuation components are summarized in Table 1.

## 3. Modeling and Simulation

### 3.1. Dynamic Simulation

**Figure 3:**
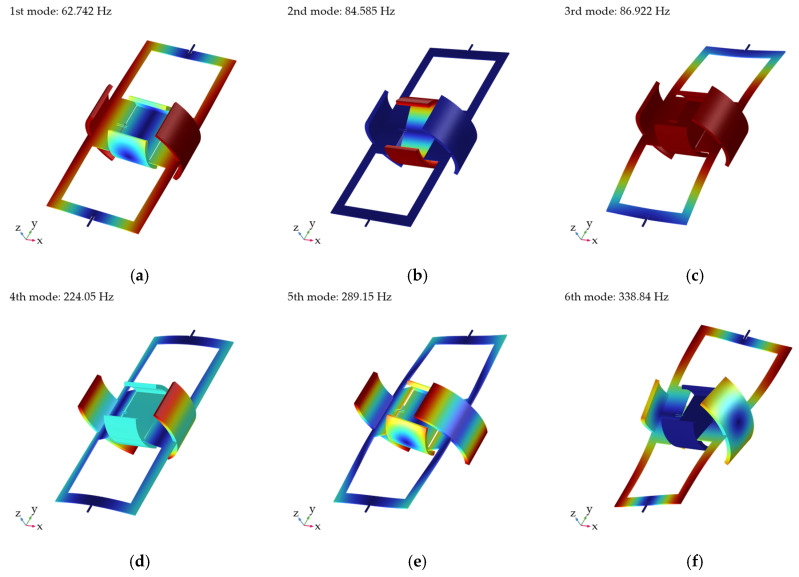
Resonant scanning modes of scanning micromirror: (**a**) 1st mode; (**b**) 2nd mode; (**c**) 3rd mode; (**d**) 4th mode; (**e**) 5th mode; (**f**) 6th mode.

Modal analysis serves as the foundation for the dynamic characterization of scanning micromirrors, and it is critical to conduct modal analysis on the mirror structure. To observe dynamic stability, the six modal frequencies of the scanning micromirror were analyzed, and the simulated results are shown in Figure 3. The densities, Young’s moduli, and Poisson’s ratios of the mirror structure and the permanent magnets are summarized in Table 2. In Figure 3a, the first mode observed was the mirror torsion mode along the y-axis at 62.747 Hz. In Figure 3b, the second mode observed was the mirror torsion mode along the x-axis at 84.585 Hz. The third mode observed was the piston mode at 86.922 Hz. The fourth mode created a twisting action at 224.05 Hz. The fifth mode and the sixth mode observed were complex—a combination of torsional and bending modes at 289.150 Hz and 338.840 Hz.

As the mirror structure was designed in non-resonant scanning mode, the minor differences between the first mode and the second mode do not affect the operational stability.

### 3.2. Optical Scanning Angle Analysis

**Figure 4:**
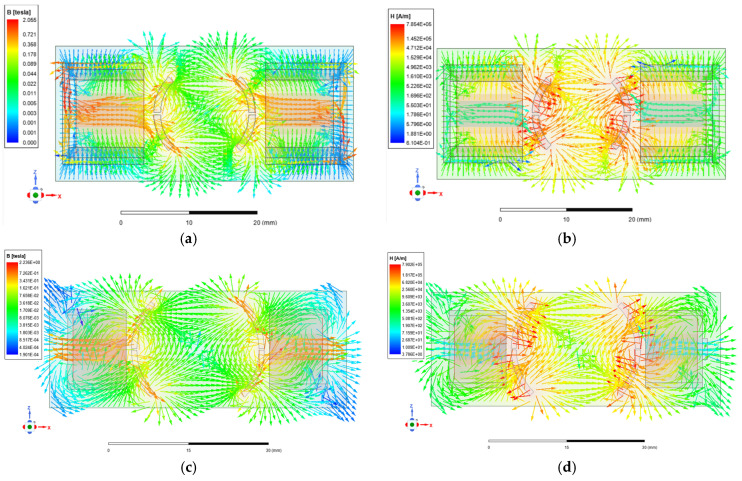
The cross-sectional distribution of (**a**) B and (**b**) H on the mirror plate. The cross-sectional distribution of (**c**) B and (**d**) H on the internal frame.

When the micromirror is in operation, the distribution characteristics of the electromagnetic field within the micromirror structure are analyzed. In this case, the magnetomotive force (MMF) is equal to the current per turn multiplied by the number of turns. When the input current is 10 mA, the magnetic field distribution of the scanning micromirror is simulated. Figure 4 illustrates the cross-sectional distributions of the magnetic flux density (B) and the magnetic field intensity (H) on the mirror plate and the internal frame.

Simulation results reveal that the maximum B on the mirror plate is concentrated near the boundaries of the iron cores, reaching approximately 2.055 T. The distribution of H indicates that regions with high H-values are concentrated near the boundaries of the permanent magnets, reaching a maximum magnitude of approximately 7.854 × 10^5^ A/m. The distribution patterns of B and H on the internal frame are similar to those on the mirror plate, with maximum values of 2.236 T and 7.902 × 10^5^ A/m.

**Figure 5:**
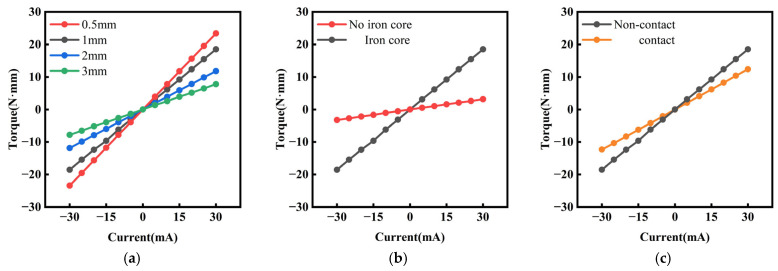
Current–torque characteristics: (**a**) under different distances; (**b**) with/without iron core; (**c**) with/without contact.

In simulations, the deflection angle of the scanning micromirror is determined by the displacement of the mirror plate and the internal frame, which is directly proportional to the magnitude of the electromagnetic torque. Thus, enhancing the deflection angle requires increasing the electromagnetic torque. This work optimized the electromagnetic torque through three design strategies:

Reducing the distance between the permanent magnets and the copper coils ($L_{mi}$). Figure 5a compares the magnitudes of electromagnetic torque at varying driving currents with distances of 0.5 mm, 1 mm, 2 mm, and 3 mm between the permanent magnets and the copper coils. The results demonstrate that smaller distances lead to higher electromagnetic torque, confirming the advantages of selecting arc-shaped permanent magnets over rectangular ones, as discussed in Section 2.

Embedding the iron cores in the copper coils. Figure 5b compares the electromagnetic torque with and without the iron cores at varying driving currents, showing that the iron cores significantly enhance the electromagnetic torque.

Maintaining non-contact between the copper coils and the iron cores. Figure 5c compares the electromagnetic torque in contact and non-contact configurations, revealing that non-contact prevents magnetic saturation and eddy current losses. The results indicate that maintaining a non-contact configuration between the copper coils and the iron cores effectively improves the electromagnetic torque.

**Figure 6:**
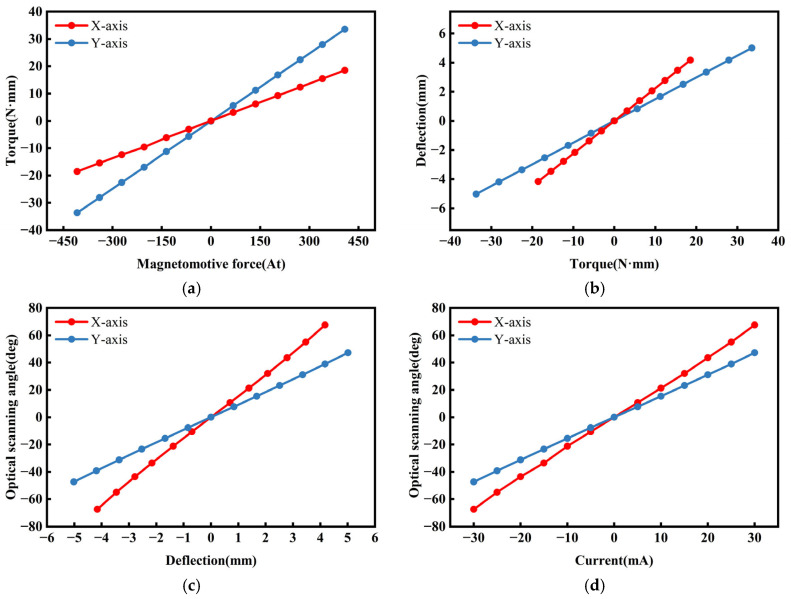
(**a**) MMF–torque characteristics. (**b**) Torque–deflection characteristics. (**c**) Deflection–optical scanning angle characteristics. (**d**) Current–optical scanning angle characteristics.

Figure 6a,b,c respectively, show the line charts of the x-axis and y-axis for the relationships between the MMF and the electromagnetic torque, the electromagnetic torque and the deflection, and the deflection and the optical scanning angle to illustrate the connections among these three parameters. Figure 6d presents the current–optical scanning angle curves.

## 4. Experiments

### 4.1. Experimental Setup

**Figure 7:**
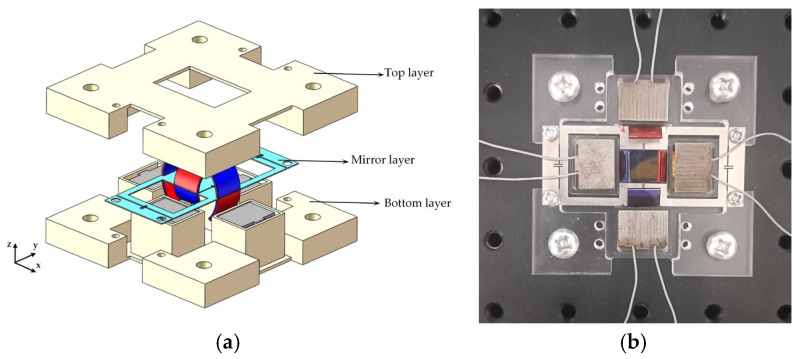
Packaging scheme of the scanning micromirror: (**a**) 3D exploded view; (**b**) photograph of the prototype.

To measure the dynamic performance of the biaxial electromagnetically driven scanning micromirror, we fabricated a prototype, as shown in Figure 7a,b. The scanning micromirror prototype has a sandwich structure: the middle layer is a stainless-steel mirror layer with dimensions of 15 mm × 10 mm. The top layer and the bottom layer are made of acrylic to isolate the copper coils from the permanent magnets and prevent the copper coils from adhering to the permanent magnets when energized. The driving part is composed of a three-part structure.

**Figure 8:**
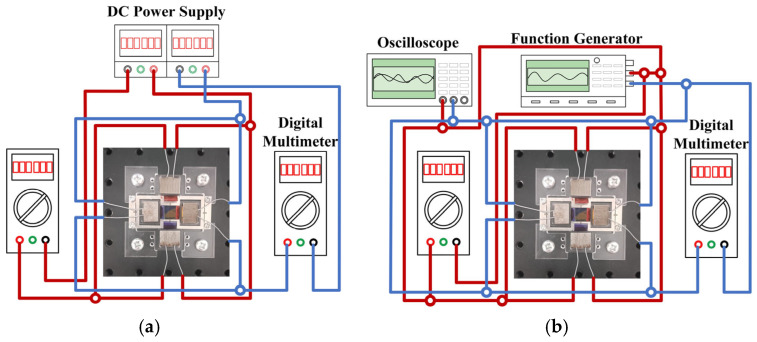
(**a**) The schematic diagram of angle measurement under non-resonant scanning mode. (**b**) The schematic diagram of frequency measurement under resonant scanning mode.

We conducted experiments using the fabricated electromagnetically driven scanning micromirror prototype to measure both the non-resonant optical scanning angle and the resonant frequency of the micromirror. For non-resonant angle measurement, the input signal was supplied by a DC power supply. Digital multimeters were used to monitor the DC current. Figure 8a shows a schematic diagram of the non-resonant optical scanning angle measurement setup, where the scanning micromirror is independently driven by the DC power supply.

During resonant frequency measurement, sinusoidal excitation signals at specific frequencies are generated by the function generator and applied to the copper coils. A digital multimeter and oscilloscope are used to measure the AC current magnitude and input signal waveform. During both non-resonant optical scanning angle and resonant frequency measurements, the scanning micromirror is independently driven along the x-axis and the y-axis. Two copper coils on the same axis are connected in parallel. Figure 8b shows a schematic diagram of the resonant frequency measurement setup, where the scanning micromirror is independently driven by the function generator.

**Figure 9:**
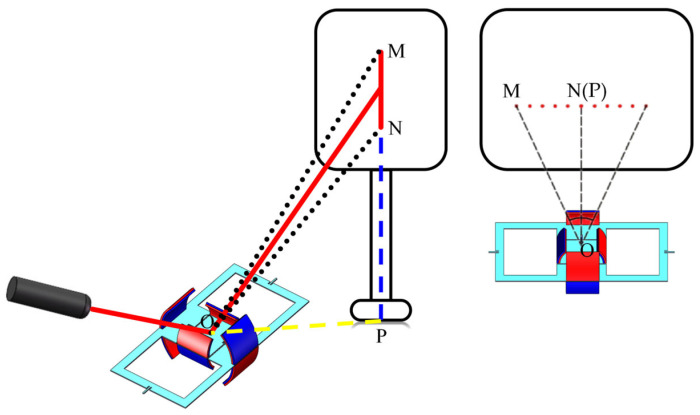
Schematic diagram of angle measurement using laser triangulation method.

A laser serves as the light input signal. After reflecting off the mirror surface, the light is received by a reception screen. The optical scanning angle is measured using the laser triangulation method shown in Figure 9. It is known that the optical scanning angle is twice the mechanical deflection angle of the scanning micromirror, and the calculation method is as follows:

(1) $$\theta_{\mathrm{OSA}}=\arctan\left(\frac{L_{\mathrm{MN}}+L_{\mathrm{NP}}}{S_{\mathrm{OP}}}\right)-\arctan\left(\frac{L_{\mathrm{NP}}}{S_{\mathrm{OP}}}\right)$$

where $L_{\mathrm{MN}}$ represents the total length of the light beam or the light spot, $L_{\mathrm{NP}}$ represents the distance from the lowest point of the light beam or the light spot to the reference point, and $S_{\mathrm{OP}}$ represents the length from the micromirror to the reference point.

### 4.2. Experimental Results

#### 4.2.1. Static Test

**Figure 10:**
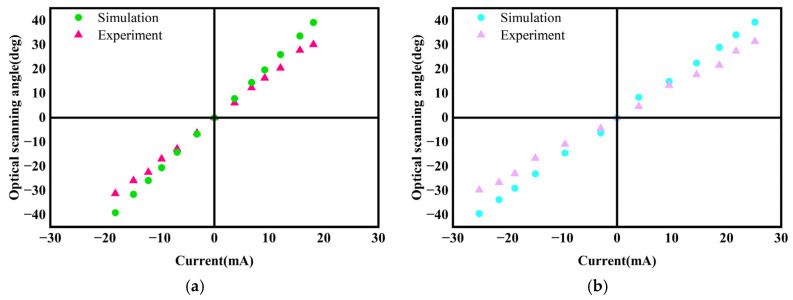
Experimental and simulation results for optical scanning angle: (**a**) x-axis; (**b**) y-axis.

The static performance of the scanning micromirror was characterized using a DC power supply, laser, reception screen, and grid paper. Current signals were applied through the DC power supply, and the laser spot positions were recorded at varying currents. Based on the optical scanning angles calculated using the laser triangulation method, relationships between input current and optical scanning angle in both the x-axis and y-axis directions were established. Experimental results were compared with simulation results. Figure 10a,b present comparison plots of simulated versus experimental data for the x-axis and y-axis directions. There is a discrepancy between the experimental results and the simulated results, primarily due to the following factors:

Assembly tolerances. During the assembly process, differences between the actual assembly positions and the simulated positions may arise due to errors in manual assembly. These discrepancies are reflected in the permanent magnet’s placement, the distance between the permanent magnet and the copper coil, and the gap between the coil and the iron core.

Coil heating. When the driving current applied to the coil increases, the copper coil may experience a rapid temperature rise in a short period. The thermal demagnetization effect may reduce the effective electromagnetic torque, leading to a decrease in the optical scanning angle.

**Figure 11:**
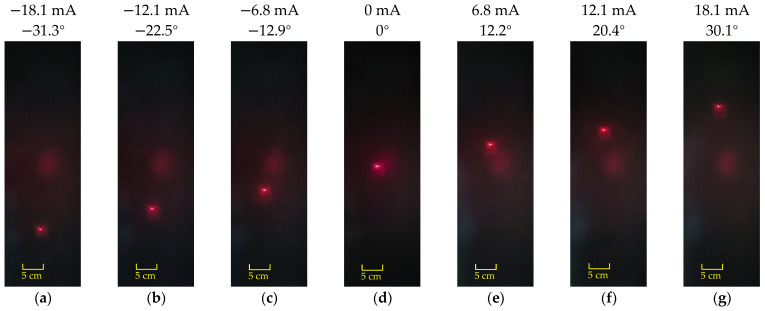
The scanning profiles of the laser spot when the scanning micromirror is driven under DC currents along the x-axis: (**a**–**c**) input reverse current; (**d**) no current input; (**e**–**g**) input forward current.

**Figure 12:**
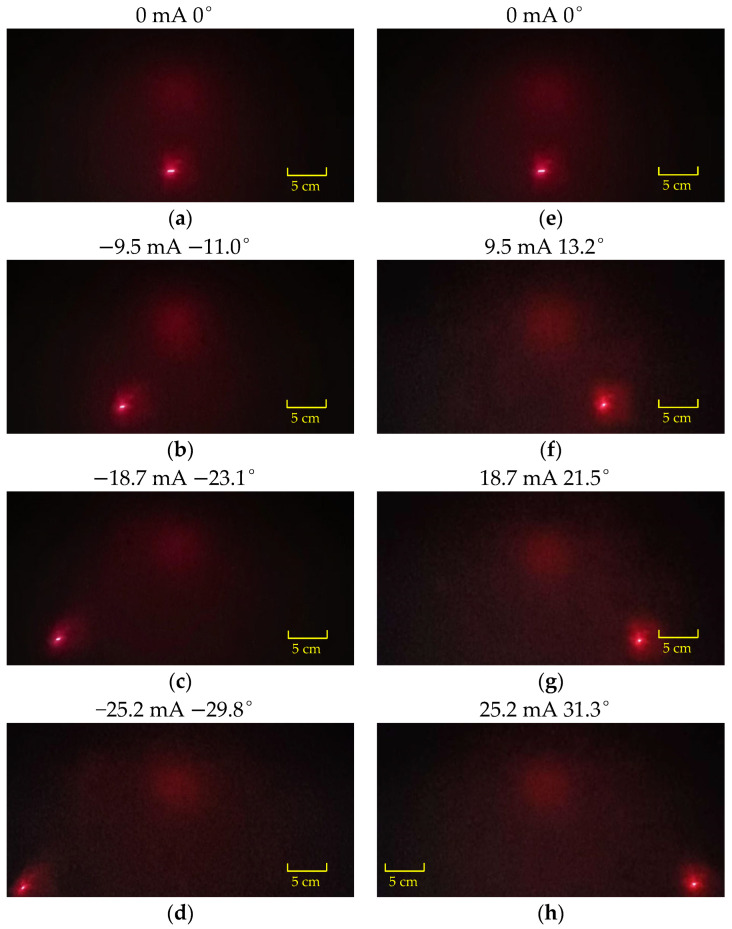
The scanning profiles of the laser spot when the scanning micromirror is driven under DC currents along the y-axis: (**a**–**d**) input reverse current; (**e**–**h**) input forward current.

Figure 11 and Figure 12, respectively, show the scanning positions of the laser spot when the scanning micromirror is driven by different DC currents along the x-axis and y-axis. Laser spot displacement analysis revealed optical scanning angles of 61.4° (x-axis) at ±18.1 mA and 61.1° (y-axis) at ±25.2 mA. Future work will explore novel approaches to address coil heating issues.

#### 4.2.2. Dynamic Test

**Figure 13:**
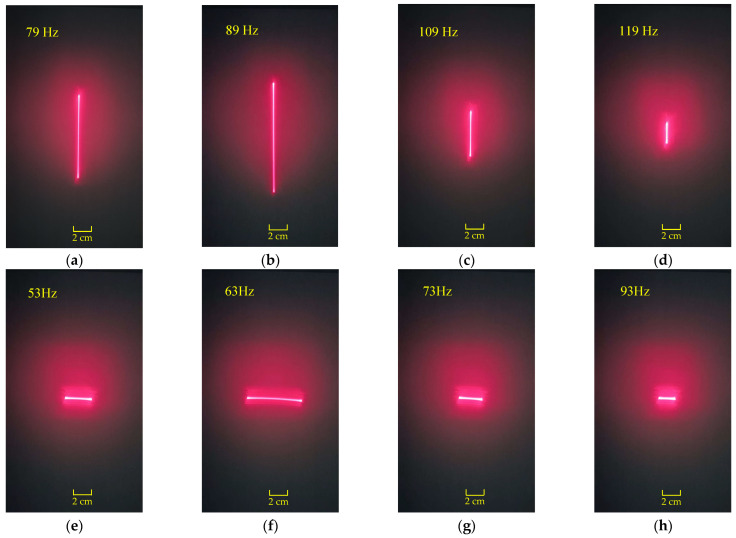
The scanning positions of the laser beam when the scanning micromirror is driven under AC currents: (**a**–**d**) along the x-axis; (**e**–**h**) along the y-axis.

The dynamic performance of the scanning micromirror was characterized using a function generator, collimated laser, reception screen, and grid paper. The experimentally measured resonant frequencies were 89 Hz along the x-axis and 63 Hz along the y-axis. Figure 13a–d show the scanning positions of the laser beam when the scanning micromirror is driven by different frequencies under 1.5 mA_rms_ along the x-axis. Figure 13e–h show the scanning positions of the laser beam when the scanning micromirror is driven by different frequencies under 1.5 mA rms along the y-axis.

**Figure 14:**
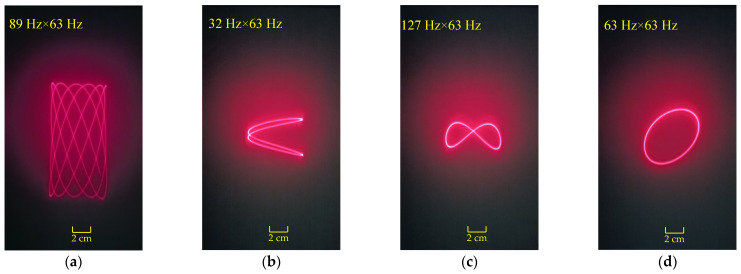

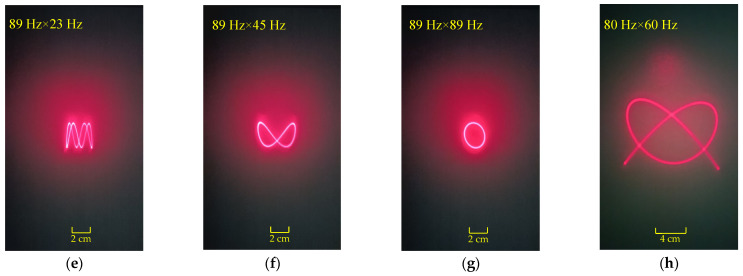
Scanning results: (**a**) biaxial resonant scanning mode, 89 Hz along the x-axis and 63 Hz along the y-axis; (**b**–**f**) uniaxial resonant and non-resonant scanning mode; (**g**,**h**) biaxial non-resonant scanning mode.

**Figure 15:**
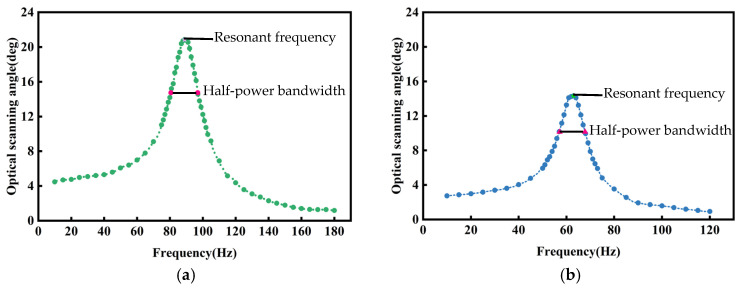
The dynamic test results of scanning micromirror with input current of 2.1 mA_rms_: (**a**) dynamic response along x-axis; (**b**) dynamic response along y-axis.

By adjusting the input signals along the two axes of the micromirror, the scanning results for three different scanning modes are shown in Figure 14. In Figure 14a–d, the input current for both axes of the micromirror is 1.5 mA_rms_. In Figure 14e–g, the input current for the x-axis of the micromirror is 0.3 mA_rms_, and the input current for the y-axis of the micromirror is 3.0 mA_rms_. In Figure 14h, the input current for both axes of the micromirror is 3.0 mA_rms_. Figure 15 shows the frequency response curves for the dual axes of the micromirror. The quality factors of the scanning micromirror are 5.48 (x-axis) and 5.92 (y-axis).

Discrepancies between experimental and simulated resonant frequencies (experiment: 89 Hz and 63 Hz; simulation: 84 Hz and 62 Hz) mainly arise due to differences in boundary constraints. During the experiments, the internal torsion beam was connected to the internal frame. The external torsional beam was connected to the external frame, which was fixed to the bottom layer using screws. During the simulations, the fixed supports of the external frame were precisely modeled. These factors caused frequency deviations between experimental and simulated modal responses.

## 5. Discussion

During both simulations and experiments, we identified key factors influencing the optical scanning angle of the micromirror in non-resonant scanning mode: coil dimensions, the distance between the copper coil and the permanent magnet, and coil heating. In the simulations, we adjusted the dimensions of the copper coil and the iron core to compare the electromagnetic torque under different conditions. The maximum torque was achieved with a copper coil external diameter of 12 mm and an internal diameter of 6 mm, which was the configuration adopted in this work. The primary reasons for this phenomenon are as follows: On the one hand, the magnetic interaction is enhanced. When the copper coil interacts with the permanent magnets, the iron core is simultaneously magnetized. By maintaining constant magnet dimensions, the introduction of the iron core significantly increases the magnetic field strength and reduces flux leakage through optimized flux distribution. On the other hand, the iron core exhibits superior magnetic permeability. The iron core’s permeability is much higher than that of air, lowering the magnetic reluctance and enhancing the utilization efficiency of the magnetic field.

Coil heating is a critical factor that affects the optical scanning angle of the micromirror in non-resonant scanning mode. On the one hand, due to the negative correlation between current density and coil internal diameter, when the current in the copper coil increases, smaller inner diameters result in a worse heat dissipation capacity, triggering non-linear growth of thermal power. On the other hand, a rapid temperature rise in the copper coil over short periods can lead to partial attenuation of the magnetic field strength and thermal demagnetization effects. This thermal demagnetization reduces the electromagnetic torque. Additionally, thermal deformation of the copper coil may distort magnetic flux line distributions, increasing flux leakage rates. In future research, we will conduct further investigations into coil heating issues and explore embedding aluminum nitride ceramic sheets between the iron core and the copper coils to reduce coil temperature rise.

## 6. Conclusions

This study presents a biaxial electromagnetically driven scanning micromirror designed to address current demands for large apertures, wide scanning angles, and low fabrication costs. The micromirror is fabricated from stainless steel and employs a moving-magnet type to achieve large non-resonant optical scanning angles of 61.4° (x-axis) at around ±18.1 mA and 61.1° (y-axis) at around ±25.2 mA. The mirror surface has dimensions of 9.54 mm × 10 mm, and the resonant frequencies are 89 Hz and 63 Hz. Additionally, this study employs low-cost CNC machining processes for manufacturing, reducing production costs and providing new insights for commercial applications of micromirrors. It has potential application prospects in fields such as projection display and optical communication. Furthermore, this work provides a reference for the applications of micromirrors in LiDAR and robot obstacle avoidance.

## Figures and Tables

**Table 1:** The dimensions of designed scanning micromirror.

Parameter		Symbol	Value	Units
Mirror plate	Length of mirror plate	$L_{m}$	15	mm
	Width of mirror plate	$W_{m}$	10	mm
	Thickness of mirror plate	$T_{m}$	300	µm
Internal torsional beam	Length of internal beam	$L_{s}$	3	mm
	Width of internal beam	$W_{s}$	0.2	mm
External torsional beam	Length of external beam	$L_{f}$	3	mm
	Width of external beam	$W_{f}$	0.4	mm
Internal permanent magnet	Internal diameter of magnet	$D_{\mathrm{iim}}$	13	mm
	External diameter of magnet	$D_{\mathrm{eim}}$	15	mm
	Thickness of magnet	$T_{\mathrm{im}}$	1	mm
	Radian of the magnet	$R_{\mathrm{im}}$	45	deg
External permanent magnet	Internal diameter of magnet	$D_{\mathrm{iem}}$	23	mm
	External diameter of magnet	$D_{\mathrm{eem}}$	25	mm
	Thickness of magnet	$T_{\mathrm{em}}$	1	mm
	Radian of the magnet	$R_{\mathrm{em}}$	45	deg
Coil	Internal diameter of coil	$D_{\mathrm{ic}}$	6	mm
	External diameter of coil	$D_{\mathrm{ec}}$	12	mm
	Thickness of coil	$T_{c}$	10	mm
	Wire diameter of coil	$D_{\mathrm{wc}}$	40	µm
	Resistance of the coil	$R_{c}$	4709	Ω
Iron core	Length of iron core	$L_{\mathrm{ic}}$	15	mm
	Width of iron core	$W_{\mathrm{ic}}$	12	mm
	Thickness of iron core	$T_{\mathrm{ic}}$	15	mm

**Table 2:** Simulated parameters of the mirror structure and the permanent magnet.

Type	Material	Young’s Modulus (GPa)	Density (kg/m^3^)	Poisson’s Ratio
Mirror plate	UNS S30400 (stainless steel)	193	7930	0.31
Permanent magnet	N52 (sintered NdFeB)	190	7500	0.24

## Data Availability

The original contributions presented in this study are included in the article. Further inquiries can be directed to the corresponding author.
